# Breastfeeding and Neonatal Age Influence Neutrophil-Driven Ontogeny of Blood Cell Populations in the First Week of Human Life

**DOI:** 10.1155/2024/1117796

**Published:** 2024-07-23

**Authors:** Sebastiano Montante, Rym Ben-Othman, Nelly Amenyogbe, Asimenia Angelidou, Anita van den Biggelaar, Bing Cai, Yixuan Chen, Alansana Darboe, Joann Diray-Arce, Rebecca Ford, Olubukola Idoko, Amy Lee, Mandy Lo, Kerry McEnaney, Mehrnoush Malek, David Martino, Geraldine Masiria, Oludare A. Odumade, William Pomat, Casey Shannon, Kinga Smolen, The EPIC Consortium, Al Ozonoff, Peter Richmond, Scott Tebbutt, Ofer Levy, Beate Kampmann, Ryan Brinkman, Tobias Kollmann

**Affiliations:** ^1^ BC Cancer Agency, 675 West 10th Avenue, Vancouver V5Z 1G1, BC, Canada; ^2^ Telethon Kids Institute Perth Children's Hospital, 15 Hospital Avenue, Nedlands 6009, WA, Australia; ^3^ RAN BioLinks Ltd., 10212 Yonge Street, 202, Richmond Hill L4C 3B6, Ontario, Canada; ^4^ Department of Microbiology and Immunology Department of Pediatrics; Dalhousie University, 6299 South Street, Halifax B3H 4R2, Canada; ^5^ Precision Vaccines Program Department of Pediatrics Boston Children's Hospital, Boston, MA, USA; ^6^ Department of Neonatology Beth Israel Deaconess Medical Center, 330 Brookline Avenue, Boston, MA 02215, USA; ^7^ Harvard Medical School, Boston, MA, USA; ^8^ Wesfarmers Centre of Vaccines and Infectious Diseases Telethon Kids Institute University of Western Australia Perth, 15 Hospital Avenue, Nedlands, WA 6009, Australia; ^9^ Department of Pediatrics BC Children's Hospital University of British Columbia, 4480 Oak Street, Vancouver V6H 3V4, BC, Canada; ^10^ Vaccines and Immunity Theme Medical Research Council Unit The Gambia at the London School of Hygiene and Tropical Medicine, Atlantic Boulevard, Banjul P.O. Box 273, Gambia; ^11^ Papua New Guinea Institute of Medical Research, Homate Street, 441, Goroka, Eastern Highlands Province, Papua New Guinea; ^12^ Department of Clinical Research Faculty of Infectious and Tropical Diseases London School of Hygiene and Tropical Medicine, London WC1E 7HT, UK; ^13^ Department of Molecular Biology and Biochemistry Simon Fraser University, 8888 University Dr. Burnaby V5A1S6, Burnaby, British Columbia, Canada; ^14^ Wal-yan Respiratory Research Centre Telethon Kids Institute University of Western Australia, Perth, Australia; ^15^ PROOF Centre of Excellence, 10th floor, 1190 Hornby Street, Vancouver V6Z 2K5, British Columbia, Canada; ^16^ UBC Centre for Heart Lung Innovation St. Paul's Hospital, 1081 Burrard Street, Vancouver, British Columbia V6Z 1Y6, Canada; ^17^ NIH, National Institute of Health, Bethesda, USA; ^18^ Broad Institute of MIT and Harvard, Cambridge, MA, USA; ^19^ Division of Pediatrics School of Medicine University of Western Australia, 35 Stirling Highway, Crawley 6009, WA, Australia; ^20^ Centre for Global Health and Institute for International Health Charite Universitatsmedizin, Berlin, Germany; ^21^ Department of Medical Genetics University of British Columbia, 675 West 10th Avenue, Vancouver, British Columbia V6T1Z4, Canada; ^22^ Microbiology and Immunology Pediatric Infectious Diseases Dalhousie University, CEO, Born, Strong Initiative, Halifax, Nova Scotia, Canada

## Abstract

The first few days of life are characterized by rapid external and internal changes that require substantial immune system adaptations. Despite growing evidence of the impact of this period on lifelong immune health, this period remains largely uncharted. To identify factors that may impact the trajectory of immune development, we conducted stringently standardized, high-throughput phenotyping of peripheral white blood cell (WBC) populations from 796 newborns across two distinct cohorts (The Gambia, West Africa; Papua New Guinea, Melanesia) in the framework of a Human Immunology Project Consortium (HIPC) study. Samples were collected twice from each newborn during the first week of life, first at Day of Life 0 (at birth) and then subsequently at Day of Life 1, 3, or 7 depending on the randomization group the newborn belongs to. The subsequent analysis was conducted at an unprecedented level of detail using flow cytometry and an unbiased automated gating algorithm. The results showed that WBC composition in peripheral blood changes along patterns highly conserved across populations and environments. Changes across days of life were most pronounced in the innate myeloid compartment. Breastfeeding, and at a smaller scale neonatal vaccination, were associated with changes in peripheral blood neutrophil and monocyte cell counts. Our results suggest a common trajectory of immune development in newborns and possible association with timing of breastfeeding initiation, which may contribute to immune-mediated protection from infection in early life. These data begin to outline a specific window of opportunity for interventions that could deliberately direct WBC composition, and with that, immune trajectory and thus ontogeny in early life. This trial is registered with NCT03246230.

## 1. Introduction

Infectious diseases disproportionately burden the very young in low-resource settings and constitute a major cause of early childhood death around the world [1, 2, 3, 4]. Protection from infection is provided at least in part by the immune system, which changes rapidly over the first few hours to days of life [5]. Ontogeny encompasses the development of an individual from conception to death and plays a critical role in shaping an organism's biology, including the immune system [6, 7]. Neonatal immune ontogeny can be influenced by factors such as gestational age at birth, with distinct immune trajectories arising from circumstances impacting pregnancy and delivery [8, 9]. Additionally, vaccination and nutrition, particularly early initiation of breastfeeding within the first hour of life, are presumed to modulate newborn immune development [10, 11, 12, 13, 14]. But how vaccination and or breastfeeding impacts early life immune ontogeny is not sufficiently well delineated to guide interventions [15]. For example, the impact of vaccination on neonatal immune development has not been delineated beyond the pathogen-specific immune responses (e.g., antibodies to the vaccine target) [5]. Equally unknown is how colostrum, the first milk produced by the mother, and known to be rich in immune factors provides enhanced protection from infection for newborns remains unclear [16, 17].

To address this knowledge gap, we conducted a study with the objective to capture changes in white blood cell (WBC) composition in two diverse populations in low-resourced settings, The Gambia in West Africa and Papua New Guinea in Australasia as part of the Expanded Program on Immunization Consortium (EPIC) [18]. We hypothesized that internal (e.g., age) as well as external (e.g., vaccination, feeding) factors combine to drive immune ontogeny across the first week of life. We set out to capture the impact of these variables (age, vaccination, breastfeeding) on immune ontogeny using multiparameter flow cytometry and novel computational tools that enable high-end analysis using only minimal amounts of peripheral venous blood [19, 20]. Our findings confirm that the first week of human life is indeed a highly dynamic period but one that follows common immune trajectories across different populations and geographies [21]. We also identified that breastfeeding and immunization significantly influence age-dependent changes in neutrophil and monocyte composition, while factors such as sex, maternal age at birth, and birth season had minimal impact. This begins to identify a window of opportunity for optimizing interventions like breastfeeding and vaccination to shape favorable immune trajectories in newborns.

## 2. Materials and Methods

### 2.1. Study Participants

In total, 796 healthy term newborns were recruited from two distinct geographical regions: The Gambia in West Africa and Papua New Guinea (PNG; Eastern Highland province) in Melanesia. Recruitment took place at three different locations: The Kanifing General Hospital and The Banjullinding Health Center in the Gambia, Goroka General Hospital in PNG. The newborns were assigned to two distinct cohorts based on enrollment periods: a primary cohort comprising 711 newborns exclusively from The Gambia (main cohort), and a smaller secondary cohort consisting of 85 newborns, with 40 from PNG and 45 from The Gambia. This resulted in a final participant count of 756 from The Gambia and 40 from Papua New Guinea (PNG), who were enrolled between September 2017 and August 2022. The secondary, smaller cohort started midway through the study period. Inclusion criteria require the participant to be a healthy infant under 24 hr of age born naturally from mothers at a gestational age over 37 weeks, who is unexposed to HIV and with a weight of 2.5 kg or greater at the time of enrollment. Exclusion criteria disallow participation if the infant is born premature, febrile, has unstable vital signs with an Apgar score <8 or born from a mother who is HIV-positive or exposed or has hepatitis B antigens. Mothers using antibiotics in the week prior delivery or diagnosed with tuberculosis in the past 6 months were also excluded. The type of birth delivery (natural or C-section) was also considered, and women were retained in the study if they had spontaneous vaginal delivery. Nevertheless, nearly all babies were born naturally, which is the prevalent mode of birth in both regions. Written informed consent was obtained from parents or caretakers of all newborns prior to their participation in the study. In particular, women were informed about the study when presenting for antenatal care in the second or third trimester of pregnancy. They were given information regarding the study using a detailed informed consent document. Individuals expressing interest in participating were provided with a copy of the informed consent document to take home to discuss with their partners or other significant decision makers in the family. When the mother of the potential participant presented in labor or shortly following delivery, the consent signature was obtained, and eligibility was assessed. Maternal infant pairs were then recruited within the first 24 hr of the infants' life. If there were no complications, maternal infant pairs were usually sent home within 24–48 hr postdelivery. There were no sample collections carried out at home. However, field staff conducted a home visit during the first week of life to evaluate for sick infants, such that all participants had a total of two visits (home or clinic) prior to day 7 of life.

In the main cohort, newborns were randomized to vaccine groups as previously described, including a subset of newborns who did not receive any vaccine until Day of Life (DOL) 7 [18]; all newborns in the secondary cohort were vaccinated on DOL7. The study is registered with Clinicaltrials.gov under registration number NCT03246230. Ethical approvals have been obtained for the core study in The Gambia from The Gambia Government/MRCG Joint Ethics Committee (Scientific Coordinating Committee number: 1513) and for the validation study in PNG from the PNGIMR Institute Review Board (IRB number: 18.12). The protocol has been approved by the PNG Medical Research Advisory Committee (IRB number 18.14). Ethical approval has also been obtained from the Boston Children's Hospital Institutional Review Board (IRB-P00024239).

### 2.2. Clinical Data Description and Data Availability

Our study involved the collection of a wide range of participant data, including demographics and study visit information. We also recorded the time of sample collection relative to birth and each participant's date and time of birth. The breastfeeding status of the participants was captured by focusing on the concept of “delayed breastfeeding,” which is not part of the study procedures nor objectives but denotes the duration after birth that a newborn went without breastfeeding. In particular, the study nurses visited the mother starting within 12 hr of birth. During these visits, the nurses asked the mother if she had breastfed the baby since delivery and recorded the response as either “Yes” or “No” on the study forms. This approach categorized the baby as either breastfed or not in the period preceding the question being asked. Given the timing of the visit in relation to birth depending on factors such as whether the birth occurred during the day or night, we were able to capture a wide range of time intervals that allowed our approach to stratify the initiation of breastfeeding. All mothers were encouraged to initiate breastfeeding during these interactions. All women at both of our sites were counseled to breastfeed as soon as possible postdelivery and at each postnatal visit following the World Health Organization (WHO) protocol [22]. Other essential variables such as newborn weight, vaccination group, sex, time of birth, and maternal age at delivery were also recorded. Vaccination groups include administration of only Bacille Calmette–Guérin (BCG), only Hepatitis B vaccine (HBV), both vaccines, or no vaccine at birth (i.e., within the first 24 hr of life). The age of the newborn at each visit (in hours) was also documented. This comprehensive dataset underpinned our subsequent analyses. All deidentified data from this study are accessible in ImmPort with the accession numbers SDY1538 and SDY2311, as previously reported [18]. The clinical data were collected using REDCap with information obtained via questionnaires as well as medical records. All data were collected using electronic case report forms (CRFs) with built-in quality checks designed on REDCap except for consent forms and certified copies of medical records. Data were checked for congruency and completeness at the site and quality control (QC) checks were completed by data assistants and a data manager at both sites. Table S1 summarizes the main clinical attributes of mothers and newborns, comparing the clinical information available between Gambian and PNG subjects.

### 2.3. Participant Sampling and Blood Processing

Within the first week of life, newborns were sampled twice to collect peripheral blood for analysis. One sample was collected at birth (Day of Life 0, DOL0) and a follow-up blood sample was collected at either DOL1 or 3 or 7. For each phlebotomy, 2 ml of peripheral blood was collected by venipuncture in heparinized tubes. Fresh blood specimens were further processed on site, preserved in appropriate buffers, and saved at −80°C for downstream analysis, as described [21]. Briefly, 225 *μ*l of whole blood cells mixed with an equal volume of culture medium (RPMI Glutamax) were stained with fixable viability dye (eBioscience, USA) prior to fixation and stored in Smart tube solutions at −80°C (Smart Tube, CA, USA). Samples were shipped on dry ice under controlled temperatures and monitored conditions (World Courier, USA).

### 2.4. Immune Phenotyping Sample Quality Control and Manual Analysis

Flow cytometry samples were thawed in a water bath at 10°C and separated by centrifugation at 600 g for 10 min. Cell pellets were washed by centrifugation in phosphate buffered saline (PBS) and stained per manufacturer recommendations. Two flow cytometry panels with 13 surface markers each were designed to identify adaptive and innate cell populations (including neutrophils, dendritic cells (DCs), natural killer (NK) cells, monocytes, basophils, T cell subsets and B cell subtypes) (Table S2). After staining, blood cells were washed in PBS, resuspended in 200 *μ*l of PBS and FCM data were acquired on a LSRII flow cytometer (BD Biosciences). Samples were run in five different batches with up to 200 samples analyzed daily in a high throughput FCM in 96 well plates resulting in 1,560 biological samples per panel from a total of 796 study participants analyzed by flow cytometry.

### 2.5. Flow Cytometry Assays and Sample Quality Control

Initial quality control (QC) was performed through visual inspection of the compensated FCM data using FlowJo software (version 9.9, Becton Dickinson, USA) to ensure the experimental conditions were optimal and all major populations could be identified according to predefined gating strategies (Figures S1 and S2). Five QC steps were performed on the FCM data prior to final analysis:

- Sample quality: At thawing, blood samples were inspected for color and volume. After centrifugation cell pellets were assessed for color and size as well. For example, if the blood sample was coagulated or dark red and/or the cell pellet was observed red, this indicated that red blood cell lysis was incomplete and the mixing with the lysing and fixing buffers prior to freezing were not optimal. These samples were flagged (four samples in the Adaptive Panel, eight in the Innate Panel), and the electronic records for those specific samples were inspected for any reported deviations to protocol prior to analysis. Samples with cell pellets not optimally resuspended (e.g., appearing very sticky even after resuspension in the saline buffer) were excluded before staining to avoid a flow cytometer clogging at the time of analysis (*n* = 4).
- Cytometer performance: CS&T beads (BD cat # 656504) were used for instrument QC to characterize, track, and report performance measurements of the cytometer according to manufacturer's instructions.
- Laser stability: Standardization beads (or Rainbow beads, Beckman Coulter, Cat # A69184) were used according to the manufacturer's instructions. The same beads were analyzed prior to the biological samples and the value for each fluorochrome was adjusted in every experiment if different from the value set in the first.
- Treatment group randomization within plates: FCM samples run in the same plate were randomized using a block randomization scheme that took into consideration the different study treatment groups and the study participant longitudinal sampling where all groups are evenly distributed across all flow cytometry analysis plates.
- Batch effects: A biological internal control was prepared according to study protocol from a biological sample prepared at the beginning of the study from a single individual and dividing the pooled sample into individual aliquots frozen at −80°C. For each experiment, a single aliquot is thawed and stained in parallel with the study samples and run in the same plate. Stability of the frozen control over time was evaluated by inspecting the linear plot of the counts of the control populations run in each plate (Figures S3 and S4). The control counts of all populations across all plates showed no statistical difference (*p*  > 0.05, Kruskal–Wallis test) (Figure S5).

All flagged samples and aliquots were still gated and analyzed during the postgating quality checking. The postgating QC steps described below determined the final samples to exclude.

### 2.6. Flow Cytometry Automated Gating and Data Quality Control (QC)

To reduce time and subjectivity associated with manual gating of FCM data, an automated data analysis pipeline was applied including time vs. fluorescence anomaly removal (flowCut) [23, 24], automated gating (flowDensity) [19], and a novel biomarker discovery algorithm (flowTypeFilter) and biomarker visualization (RchyOptimyx) [20, 25]. To this end, the data were first compensated according to a manually provided compensation matrix and transformed using the estimate_Logical function of the flowCore package [26]. Poor quality events generated by technical artifacts during acquisition were then removed using flowCut [23, 24]. Next, flowDensity performed the automated cell population identification, parameterized for each panel according to a predefined gating strategy [19]. This predefined set of populations included 33 populations in the Innate Panel and 23 populations in the Adaptive Panel. Poor-quality samples and outliers were identified and removed using four criteria. The first criterion excluded samples where the number of cells is insufficient to perform a correct complete gating due to the presence of empty or unusually small parent populations. The second criterion excluded samples with an unusual cell distribution affecting at least one population (e.g., cases in which the debris is not clearly separable from the cells). The first and second criteria were applied by visually inspecting the plots generated by the automated gating pipeline. Figures S6 and S7 show, respectively, a sample excluded based on the first and second criteria. The third criterion excludes samples with low cell counts affecting the “Size” gate (where the cells are selected based on the size and the granularity). For this criterion, the threshold of exclusion (i.e., minimum acceptable total number of cells) was calculated as the mean minus three times the standard deviation (19,000 events for the Innate Panel and 25,202 cells for the Adaptive Panel). The fourth criterion excludes the outlier samples with a low number of live cells. The presence of too many dead cells suggests a problem during the handling of the sample. In this case, the threshold of exclusion was also equal to the mean minus three standard deviations (13,000 cells for the Innate Panel and 18,805 cells for the Adaptive Panel). Finally, samples with sex mismatch in metadata identified using the RNAseq data were excluded from further analysis (metadata quality checking). A total of 781 participants (Adaptive panel) or 782 participants (Innate panel) passed this QC step and were used for further analysis (Tables S3 and S4). First, flowTypeFilter generated all possible combinations of markers (i.e., all possible immunophenotypes) based on the threshold determined by flowDensity (automated unsupervised analysis) (Figure S8), resulting in 83,385 populations in the Innate Panel and 51,195 populations in the Adaptive Panel. flowTypeFilter improves the flowType algorithm via the new function point.in.polygon of the sp package [27] to allow for determination of each cell to be “inside” or “outside” of any polygon gates in each partition strategy (Figure S9). Performance-critical parts written in C++ code and functions from the Rcpp package [28] were also implemented in the new flowTypeFilter algorithm to overcome the memory and CPU speed limit that came with the new filter functions within the subset generating iterations. To reduce false positive results, flowTypFilter populations with low cells/ul were excluded from further analysis. This QC approach removed populations with less than 10 cells/ul in the Adaptive Panel and less than 20 cells/ul in the Innate Panel. The flowTypeFilter set that passed this stringent QC included 2,638 populations in the Adaptive Panel and 2,040 populations in the Innate Panel. Finally, to estimate the number of cells in 1 ul of blood (cells/ul), cell counts of all gated populations were normalized using the number of true counting beads (Life Technology, USA). The flowTypeFilter set was further analyzed using RchyOptimyx [20, 25].

### 2.7. Data Analysis and Statistics

Principal component analysis (PCA) [29] and principal variance component analysis (PVCA) [30] were applied on the dataset generated using the flowDensity algorithm (supervised bivariate gating) to identify, respectively, the cell populations and the clinical factors with the highest variance in the data before delving deeper into the statistical analysis of ontogeny trajectories. PCA was also applied on the high-dimensional dataset generated by the flowTypeFilter algorithm (unsupervised bivariate gating) to identify the combination of markers with the highest variance in the data. The analysis of statistical differences across the time points (age in days or hours) was conducted using a Kruskal–Wallis test (when more than two time points were compared, *p* ≤ 0.05), a Wilcoxon rank-sum test (two time points with unpaired data, *p* ≤ 0.05) or a Wilcoxon Signed-Rank Test (two time points with paired data indexing samples with same subjects, *p* ≤ 0.05) [31]. The *p*-values were adjusted using Benjamini–Hochberg (BH) method [32]. The Wilcoxon rank-sum test was also applied for the comparison of two clinical subgroups at a given time point (e.g., males vs. females at DOL0). *p*-Values between 0.01 and 0.05 were considered moderately significant, *p*-values less than 0.01 were considered highly significant. The Cohen's d coefficient was used as standardized effect size to determine the directionality of the differences in cell counts between time points and clinical subgroups [33, 34]. A positive coefficient indicates a mean decreasing between two groups whereas a negative coefficient indicates a mean increasing. A higher Cohen's d absolute value indicates a higher effect size. The Cohen's d coefficient and fold change (FC) between time points were calculated to investigate the impact of breastfeeding on the first week of life, analyzing a “Delayed breastfed group” of infants who were not breastfed for varying intervals after birth, i.e., 0–3, 3–5, or 5–23 hr. The specific range of each interval was chosen to assure a similar n (number of samples) across the intervals considered.

Our study involved the comparison of clinical groups with large differences in the sample sizes. When possible, by choosing appropriate thresholds of separation, we organized our clinical groups in such a way that the number of samples in each group was similar (e.g., in the hours analysis or breastfeeding analysis). Where this was not possible, we used the Wilcoxon rank-sum test as it retains statistical power even with unequal sample sizes [35, 36]. Categorical analysis was supported by a regression analysis of data treated as a continuous variable. Furthermore, when analyzing two groups with very large differences in size, we performed multiple simulations by extracting a random part of the larger clinical group. We then applied the statistical test between each extracted part and the smaller clinical group. For example, in the comparison between Gambian and PNG participants with a difference of >1,000 samples between the two groups we performed 50 simulations to support statistical testing, extracting 70 random samples from the Gambian group to match the number of PNG samples to assure the consistency of the *p*-values across all simulations.

The breastfeeding statistical analysis was conducted only on a subgroup of newborns. As previously described, the exact time of breastfeeding was not recorded and was not part of the study design. We limited our analysis to women who answered “No” to the question: “Did you initiate breastfeeding?” In cases where breastfeeding had not yet commenced, this was recorded as “No,” and a timestamp was recorded. Thus, in order to investigate the impact of breastfeeding on the first week of life, we analyzed a subgroup of infants (defined as “Delayed breastfed group”) who were not breastfed for varying intervals after birth as previously mentioned. The analysis of the demographic factors (e.g., sex, ethnicity, or maternal age) and the analysis of vaccination impact were conducted on the whole cohort. Maternal age of 30 years was chosen as the stratification point because it represented a natural division in the age range of the enrolled mothers, with a relatively balanced sample size of mothers both below and above 30 years, thus retaining the statistical power of the analysis.

### 2.8. Data Quality Assurance (QA)

The EPIC-HIPC study established a centralized data management and analysis core (DMAC) to provide project-wide, secure data management infrastructure across clinical sites and cores. This enabled a cloud-based scientific environment for cross-platform bioinformatics and integrative analyses, as well as EPIC-HIPC-wide data QA policies and standards for each data source [37]. For data quality assessments, we conducted quality control steps including internal quality checks specific to each assay, examination of assay-clinical data concordance, missing value assessment, sex determination checks, imputation for missing values, and feature filtering. These measurements ensured the reliability and accuracy of the data for subsequent analyses in the study.

### 2.9. Data Deposition

The data analyzed in this manuscript are available at ImmPort under accession numbers SDY1538 and SDY2311. All analysis codes have been deposited at https://bitbucket.org/pvp-dmac/epic_hipc_ontogeny/src/master/Flow_Cytometry and are publicly available. flowTypeFilter code and its documentation is available on GitHub under the MIT license at https://github.com/eve-chen97/flowTypeFilter.

## 3. Results

### 3.1. Evolution of Immune Cell Populations in Newborns during the First Week of Life Was Dynamic and Primarily Driven by Innate Immune Cells of the Myeloid Lineage

**Figure 1:**
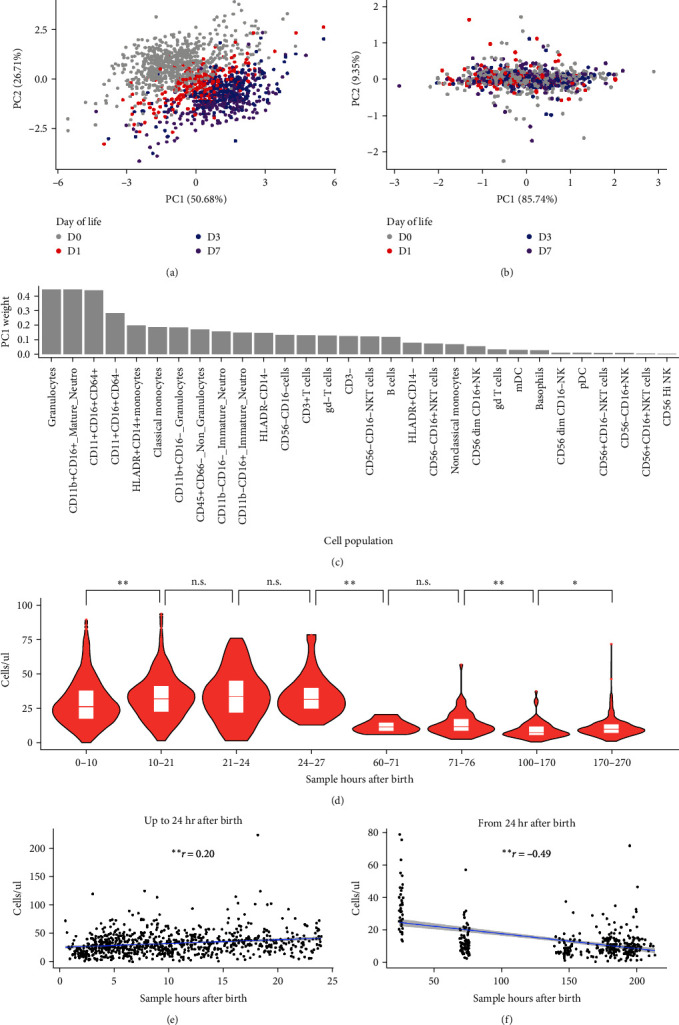
PCA analysis demonstrates age-dependent separation of innate cell populations across the first day of life. PCAs are depicted for (a) Innate Panel and (b) Adaptive Panel; (c) weights of PC1 for innate panel; (d) mature neutrophils count across time after birth, in hours. (e) Correlation between mature neutrophils counts and sample hours after birth (up to 24 hr after birth); (f) correlation between mature neutrophils counts and sample hours after birth (from 24 hr after birth).  ^*∗∗*^*p* ≤ 0.01,  ^*∗*^*p* ≤ 0.05, and n.s. *p* > 0.05 by Wilcoxon rank-sum test (d) and pearson correlation test (e, f); *r* = Pearson correlation coefficient.

PCA applied to the predefined gating approach on 1,507 samples from The Gambia and PNG indicated that the peripheral blood cell counts at birth (DOL0) were distinct from those at DOL1, 3, and 7 (Figures 1(a) and S2). Innate immune cell profiles were similar between DOL3 and DOL7, as these two time points clustered together in the PCA analysis. There was no clear clustering based on DOL in the Adaptive Panel capturing cell subsets of mostly lymphoid origin (Figures 1(b) and S1). Myeloid lineage subtypes (mature neutrophils, CD64+, and CD64−) were the main cell populations that explained the variability between different DOL, followed by monocytes, immature neutrophils and T cells (Figure 1(c)). The changes were dynamic and involved hourly fluctuations as a function of participant age. Pairwise comparison across hourly intervals showed that granulocyte counts rose significantly shortly after birth (*p* ≤ 0.01, Wilcoxon rank-sum test), reaching a peak around 10 hr postbirth before declining after the first 24 hr (*p* ≤ 0.01, Wilcoxon rank-sum test) and stabilizing to similar counts from 60 to 270 hr after birth onwards (DOL3–DOL7) (Figure 1(d)). The Pearson correlation analysis showed similar results (Figures 1(e) and 1(f)).

**Figure 2:**
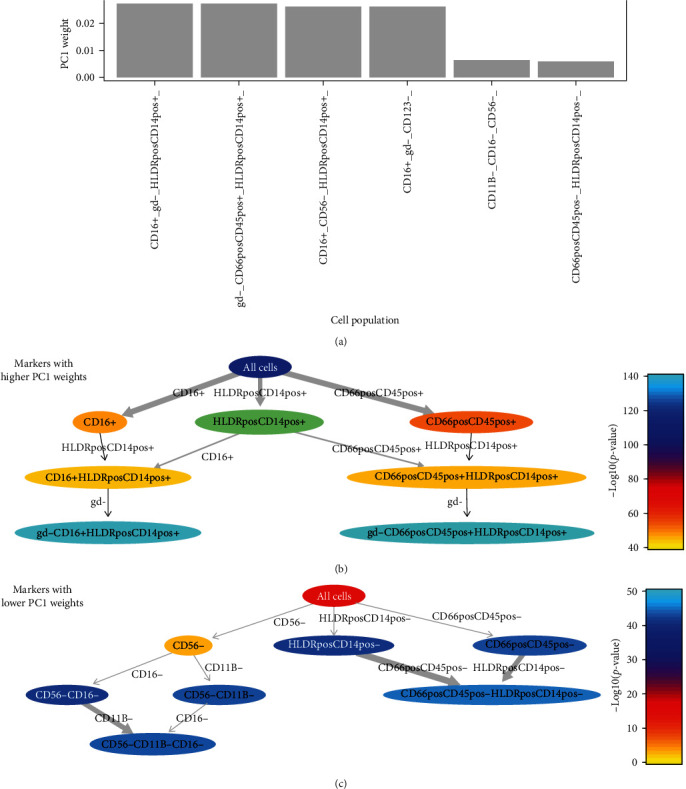
PCA analysis considering flowTypeFilter populations of Innate Panel. Weights of PC1 for Innate Panel. (a) Rchyoptymyx analysis for Innate Panel (b, c) with significant immunophenotypes colored in orange–red, not significant immunophenotypes colored in blue. The + sign after two markers (e.g., CD66posCD45pos+) indicates cells within the bivariate filter (cells within the CD66+CD45+ population filter). The + or − after one marker (e.g., CD16+, gd−) indicate the level of expression of that marker (respectively, positive expression of the CD16 marker or negative expression of the gd marker).

The PCA analysis of the unbiased approach, i.e., all possible combinations of markers (flowTypeFilter immunophenotypes) supported the results obtained from the predefined gating strategy, indicating that the neutrophil and monocyte markers contributed most to change over the first week of human life. In particular, gamma delta negative (*γδ*−) cells expressing CD16, HLA-DR, CD14, or a combination of CD45 and CD66 were the major sources of age-dependent variation in blood composition over the first week of life considering all possible combinations of markers in the Innate Panel (Figure 2(a)). CD66 and CD10 contributed most to age-dependent changes in the Adaptive Panel (Figure S10(a)). Statistical analysis across all time points (*p* ≤ 0.05, Kruskal–Wallis test) confirmed the unbiased PCA results with CD66, CD45, and CD16 markers contributing the most significant changes over time (negative log *p*-value > 100, orange-red color) (Figures 2(b) and 2(c)). These markers identify the granulocytic populations and represent the top PC1 weights observed in the Innate Panel (Figure 2(a)). CD14 and HLA-DR, the main markers used to identify monocytic populations in the Innate Panel, showed lower significance (green color, negative log *p*-value between 80 and 100). Statistical analysis of the Adaptive Panel confirmed the corresponding PCA results, with the CD66 and CD10 markers displaying the most significant changes as a function of age (Figures S10(a) and S10(c)).

**Figure 3:**
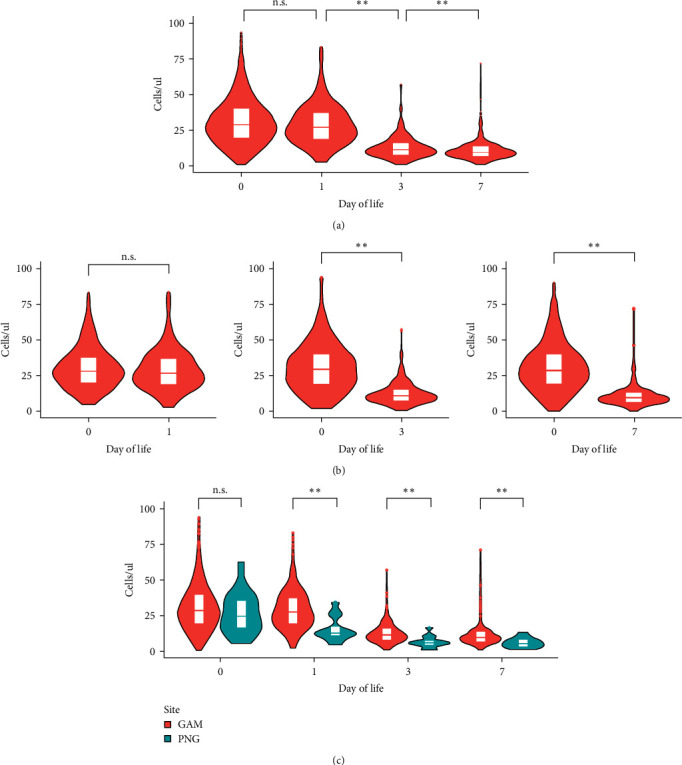
Peripheral blood neutrophil counts drop across the first week of life in two geographically distinct cohorts. (a) Mature neutrophils across the first week of life; (b) paired analysis of mature neutrophils considering same study participants comparing Visit 1 at birth (DOL0) and Visit 2 (DOL 1, 3, or 7); (c) comparison of mature neutrophils between Gambia (GAM) and Papua New Guinea (PNG) study participants across the first week of life.  ^*∗∗*^*p* ≤ 0.01 and n.s. *p* > 0.05 by Wilcoxon rank-sum test (a, c) and Wilcoxon signed-rank test (b).

Because granulocytes contributed the most to variance in cellular composition over the first week of life, we focused the analysis on the effect size and direction of change on this population. Mature neutrophils demonstrated the most dramatic and significant changes (i.e., decreasing cell counts), with a Cohen's d coefficient of 1.37 between DOL1 and DOL3 (*p* ≤ 0.01, Wilcoxon rank-sum test) (Table 1). In particular, mature neutrophil counts significantly dropped from DOL1 to DOL3 but then remained at a constant level from DOL3 to DOL7 (Figure 3(a)). This was confirmed when counts from the same study participant were used in indexed paired analysis (Wilcoxon signed-rank test) comparing neutrophil counts between DOL0 and subsequent days of life (Figure 3(b)). Nonclassical monocytes were the only innate cell populations that did not show any significant change over the first week of life (*p*  > 0.05, Wilcoxon rank-sum test). T cell subpopulations (i.e. CD3 and (*γδ* positive) on the other hand significantly increased with age (*p* ≤ 0.01, Wilcoxon rank-sum test) (Table 1).

### 3.2. Impact of Demographic Factors on Cell Distribution Over the First Week of Life

**Figure 4:**
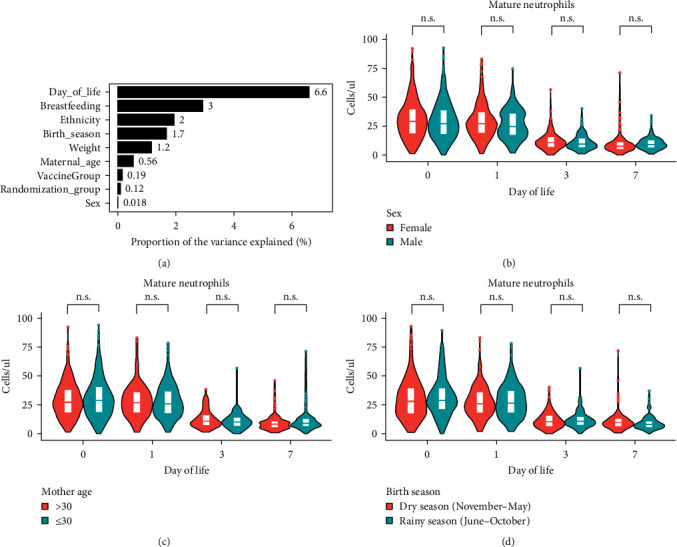
Impact of clinical factors. (a) Impact of different clinical factors on variability; (b) mature neutrophils during first week of life comparing the sex of the newborn, (c) maternal age at delivery, or (d) birth season during pregnancy. n.s. *p* > 0.05 by Wilcoxon rank-sum test.

Principal variance component analysis (PVCA) was used to investigate the impact of demographic factors on the observed immune cell changes. Age, i.e., DOL accounted for most of the variance (6.6%) followed by breastfeeding (3%) (Figure 4(a)). Among the captured demographic variables, ethnicity, birth season, birth weight, maternal age at delivery, and sex of the newborn had no relevant effect on the observed immune cell ontogeny, each accounting for <2% of variability. To further support this, no statistical differences were found in neutrophil trajectories by sex (Figure 4(b)), maternal age (Figure 4(c)), or season of birth (Figure 4(d)). Other unidentified factors not included in the clinical data captured explained the highest proportion of variance (85%) with interparticipant variations accounting only for 20% of the total variation. We did however identify a significant difference in the median counts of mature neutrophils among neonates born at different times of the day. Specifically, those born in the morning (6 am−12 pm) had significantly lower median mature neutrophil counts at birth over the first 24 hr (*p* ≤ 0.05, Wilcoxon rank-sum test) compared to those born at later times of the day. This result was confirmed using regression analysis (data not shown).

The apparent lack of ethnicity impact was surprising given the substantial differences in genetics and environment between West Africa and Melanesia. Specifically, the trajectory of neutrophils over the first week of life was compared between the different ethnic groups of Gambian newborns (*n* of each ethnicity: Wollof = 159, Serahule = 55, Mandinka = 695, Jola = 221, Fula = 176, others = 139). No significant differences by African ethnic group were found (Figures S11(a), S11(b), and S11(c)). Furthermore, the comparison between the trajectory of mature neutrophils of neonates born in The Gambia (*n* = 1,445) and those from PNG (*n* = 62) (Figure 3(c)) identified similar trajectories, i.e., showed a similar decrease in mature neutrophils after DOL1. However, the decrease in circulating neutrophil counts appeared to occur earlier for the PNG participants (*p* ≤ 0.01, Wilcoxon rank-sum test). In particular, neutrophil counts declined at DOL1 in PNG newborns while those for Gambian infants started to decline only after DOL3.

### 3.3. Breastfeeding Initiation Timing Impacts the Cell Distribution in the First Week of Life

**Figure 5:**
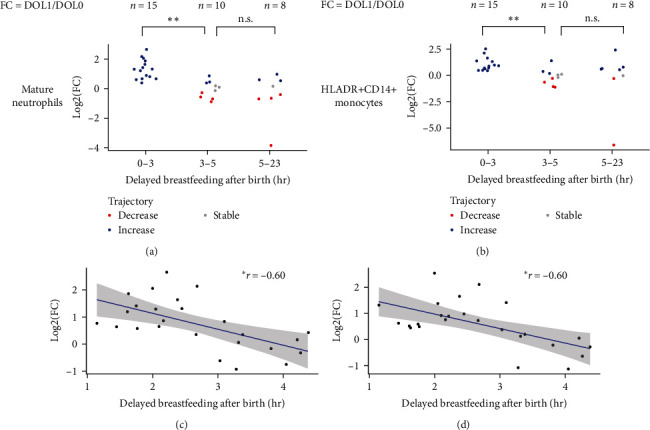
Impact of delayed breastfeeding on cell counts. Impact of delayed breastfeeding on peripheral blood mature neutrophil and HLADR+CD14+ monocyte fold change (FC = DOL1/DOL0, DOL1 = cells/ul at 1 day after day of birth, DOL0 = cells/ul at day of birth) across the first 23 hr of life (DOL0). (a) Mature neutrophils FC across time; (b) monocytes FC across time; (c) correlation between mature neutrophils FC and hours of delayed breastfeeding; (d) correlation between monocytes FC and hours of delayed breastfeeding.  ^*∗∗*^*p* ≤ 0.01,  ^*∗*^*p* ≤ 0.05, and n.s. *p* > 0.05 by Wilcoxon rank-sum test (a, b) and pearson correlation test (c, d); *r* = Pearson correlation coefficient.

Given the impact of breastfeeding on immune cell composition (3% by PVCA), we hypothesized that the time to initiation of breastfeeding after birth could be the main driver of this variability. While 87% of the newborn participants were recorded as having been breastfed “at birth,” our results identified that a longer delay of breastfeeding initiation (3–5 hr postbirth, *n* = 24) was associated with an increase in counts over the first 24 hr for NKT cells, monocytes, and granulocytes subsets (Cohen's d up to −1.500) compared to newborns for whom breastfeeding was initiated within the first 3 hr following birth (<3 hr postbirth (*n* = 28) (Table S5)). Considering the FC in cell composition between DOL0 and subsequent DOLs, we also observed a notable impact of breastfeeding on the neutrophil and monocyte cell counts starting 24 hr after birth. Earlier breastfeeding initiation (<3 hr after birth, *n* = 15) was associated with a rapid increase in neutrophil counts at DOL1, while a longer delay in breastfeeding initiation (>3−5 hr and up to 5–24 hr after birth with *n* = 10 and *n* = 8, respectively) showed no change or even a decrease in neutrophil or monocyte counts from DOL1 (Figures 5(a) and 5(b)) (Table 2). The regression analysis showed a similar trend (*p* ≤ 0.05, Pearson test, coefficient = −0.60), displaying higher FC in newborns with early initiation of breastfeeding (<3 hr) (Figures 5(c) and 5(d)). Notably, these early differences in relation to initiation of breastfeeding were not maintained to later days (DOL 3 and 7) (Figure S12).

### 3.4. Immune Cell Ontogeny during the First Week of Life Was Impacted by Vaccine(s) Given at Birth

**Figure 6:**
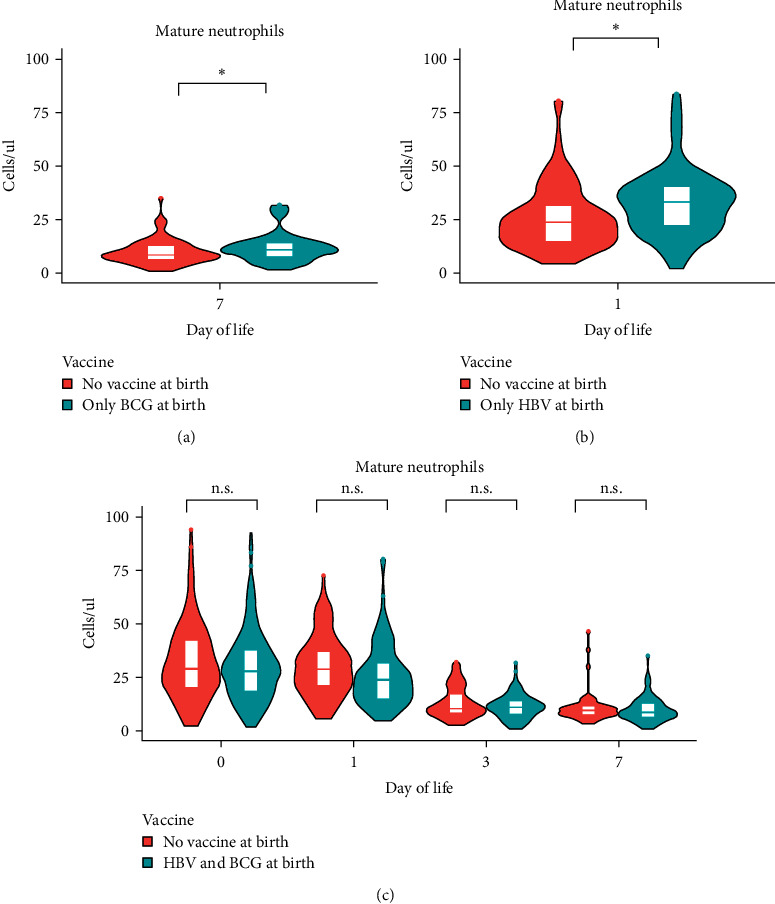
Impact of vaccines on mature neutrophil cell counts. Mature neutrophils cell counts during the first week of life comparing study participants receiving at birth (a) BCG vaccine only, (b) HBV vaccine only, and (c) both HBV and BCG vaccine.  ^*∗*^*p* ≤ 0.05 and n.s. *p* > 0.05 by Wilcoxon rank-sum test.

The impact of vaccination on immune cell ontogeny was also analyzed, particularly for the two vaccines given at birth in our cohort. Our data revealed that newborns who received the BCG vaccine at birth (*n* = 343) had a significantly higher circulating mature neutrophil count at DOL7 (*p* ≤ 0.05, Wilcoxon rank-sum test, 22% mean increase) compared to newborns who did not receive any vaccine at birth (*n* = 485) (Figure 6(a)). In newborns who received HBV at birth (*n* = 336), the neutrophil pool was significantly larger at DOL1 (*p* ≤ 0.05, Wilcoxon rank-sum test, 21% mean increase) compared to newborns with delayed vaccination (Figure 6(b)). No changes (*p*  > 0.05, Wilcoxon rank-sum test) were detected at the other time points. Simultaneous administration of both vaccines (*n* = 343) negated an impact of either vaccine administered alone, i.e., both vaccines given together did not affect changes in peripheral blood cell composition at all time points (Figure 6(c)).

## 4. Discussion

The perinatal period presents the most dramatic and rapid change of internal and external environmental conditions within the human lifespan and profoundly influences immediate as well as lifelong health [6, 38]. Despite the importance of this period, surprisingly little is known about immune development shortly after birth. Our study aimed to assess if changes of peripheral blood cell composition within the first week of life could be used to help evaluate this knowledge gap. We previously reported that contrary to the dogma of an immature, unregulated early life immune development, the newborn's first days of life follow a unique stable developmental trajectory [21]. Our new data revealed that unique neonatal immune cell developmental trajectories in peripheral blood were highly conserved across distinct populations, i.e., following a stereotypic pattern irrespective of population [7]. More precisely, the peripheral blood cell composition during the first week of life in term newborns born in distinct continents (Africa and Australasia) followed the same trajectory. We found that time of birth, time to breastfeeding initiation as well as receipt of vaccines were all significantly associated with changes in peripheral blood cell composition across the first week of life, while sex of the newborn, season of birth, maternal age, maternal environment, and/or genetic background were found not to be significantly associated.

Despite the robust, stereotypic change in peripheral blood cell composition across two distinct continents, changes appeared surprisingly fine-tuned, following not only a daily but even an hourly pattern, including differences between neonates born at different times of the day. Such variations could relate to circadian rhythm [39, 40, 41], as supported by differences in neutrophil counts during those born in the morning in our data set. While during gestation the embryonal clock is directly synchronized by the mother [42], this direct communication is lost after birth, from which point onwards the newborn relies on the development of its own independent central clock or external time signals. This would predict a gradual convergence toward a common trajectory over time, which is precisely what we observed over the first week of life.

Correlation of time to breastfeeding initiation with peripheral blood leukocyte counts suggests that early initiation of breastfeeding may play a crucial role in neonatal immune programing. The clinical relevance of “time to feed” has been demonstrated with the initiation of breastfeeding within the first hours of life providing a 2.5-fold reduction in neonatal mortality [12, 13, 14]. While the protective effect of early breastfeeding could be attributed to the transfer of colostrum, how the time to initiation of breastfeeding protects newborns against infection has not been determined [16]. To our knowledge, our data are the first to indicate a possible association of time to breastfeeding initiation with changes in immune ontogeny, thus possibly contributing to host immune-mediated protection from infection.

The analysis of peripheral blood cell composition in relation to vaccination revealed that neonates vaccinated at birth with BCG displayed higher circulating mature neutrophil counts at DOL7 compared to unvaccinated infants. Similar findings were previously reported in murine and human newborns [43, 44]. Here, we expand this observation showing that neonatal HepB vaccine administration also induces changes in neutrophil counts but earlier (DOL1). Yet, simultaneous administration of BCG and HepB vaccines negated either vaccine's individual effect. This finding suggests that there may be different mechanisms in how either vaccine mediates a change in peripheral blood neutrophil counts, and that these mechanisms may not be synergistic. Of note, simultaneous administration of BCG and HepB vaccines induces a decrease in CD3+ T cells count at DOL7 (no effect when the two vaccines are administered separately). These findings underscore the importance of considering the potential impact of vaccines on immune development in newborns and optimizing the timing of administration to maximize clinical benefit.

Our study has several limitations. For example, while similarities observed within African populations and across African–Melanesian populations suggest the existence of a common trajectory of immune cell compositional changes these patterns may not be universally generalizable. Another limitation is our focus on changes in cell composition but not functionality of the identified cells or with clinically relevant outcomes. However, absolute neutrophil counts directly correlate with the risk for serious neonatal infections [45]. It is also important to acknowledge that the precise timing of the initial breastfeeding event was not recorded; we instead used the indirect “delayed breastfeeding,” which quantifies the timelapse after birth before a newborn initiated breastfeeding. Additionally, we do not have information regarding the reason why breastfeeding was delayed such as labor conditions and degree of delay. Despite these shortcomings, our study adds valuable insights into the factors that drive composition of peripheral blood immune cells during the first week of life, emphasizing the potential significance of early breastfeeding and vaccination.

## Figures and Tables

**Table 1:** Statistical differences in cell counts over the first week of life.

Populations	Kruskal: all DOLs	Wilcoxon: DOL0,DOL1	Cohen's d: DOL0,DOL1	Wilcoxon: DOL1,DOL3	Cohen's d: DOL1,DOL3	Wilcoxon: DOL3,DOL7	Cohen's d: DOL3,DOL7
Granulocytes	0.000	0.012	0.216	0.000	**1.330**	0.016	0.184
CD45+CD66− (nongranulocytes)	0.000	0.019	−0.140	0.002	0.266	0.000	−0.595
HLADR+CD14+ Monocytes	0.000	0.149	0.127	0.003	0.289	0.000	−0.484
HLADR+CD14−	0.000	0.000	−0.338	0.706	−0.090	0.220	−0.054
HLADR−CD14−	0.000	0.004	−0.180	0.001	0.300	0.000	−0.666
Classical monocytes	0.000	0.149	0.136	0.002	0.284	0.000	−0.524
Nonclassical monocytes	0.102	0.573	0.033	0.578	0.169	0.396	0.004
mDC	0.000	0.000	−0.672	0.000	−0.828	0.000	0.391
pDC	0.000	0.000	0.382	0.002	0.321	0.000	−0.758
B cells	0.000	0.003	−0.197	0.000	0.419	0.000	−0.364
CD3+T cells	0.000	0.000	−0.297	0.001	0.308	0.000	−0.533
CD3−	0.000	0.000	0.330	0.018	0.151	0.000	−1.000
gd T cells	0.000	0.044	−0.111	0.001	0.326	0.000	−0.480
gd−T cells	0.000	0.000	−0.301	0.001	0.301	0.000	−0.525
CD56 Hi NK	0.000	0.000	0.299	0.180	−0.277	0.000	−0.636
CD56-CD16− cells	0.000	0.000	0.283	0.000	0.343	0.000	−0.320
CD56−CD16+NK	0.000	0.008	−0.182	0.039	−0.274	0.315	−0.002
CD56 dim CD16−NK	0.000	0.001	0.168	0.684	−0.080	0.000	−0.486
CD56 dim CD16+NK	0.000	0.000	0.269	0.009	0.269	0.000	−0.884
CD56+CD16+NKT cells	0.000	0.563	0.067	0.002	0.298	0.000	−0.951
CD56−CD16+NKT cells	0.000	0.043	−0.097	0.000	0.593	0.084	−0.010
CD56−CD16−NKT cells	0.000	0.000	−0.306	0.003	0.277	0.000	−0.514
CD56+CD16−NKT cells	0.000	0.083	−0.026	0.578	0.033	0.000	−0.445
Basophils	0.000	0.009	0.221	0.000	0.339	0.002	−0.137
CD11b+CD16+mature neutrophils	0.000	0.067	0.173	0.000	**1.371**	0.002	0.214
CD11b−CD16−Immature Neutrophils 1	0.000	0.000	0.453	0.000	0.450	0.000	−0.262
CD11b+CD16−granulocytes	0.000	0.000	0.423	0.000	0.413	0.153	0.026
CD11b−CD16+ Immature Neutrophils 2	0.000	0.200	0.141	0.000	1.069	0.013	0.186
CD11b+CD16+CD64+	0.000	0.149	0.148	0.000	1.045	0.000	0.338
CD11b+CD16+CD64−	0.000	0.240	0.056	0.000	0.698	0.168	−0.162

Wilcoxon rank-sum test *p*-values, Kruskal–Wallis test *p*-values, and coehn's d coefficients of all cell populations for the statistical evaluation of ontogeny changes. The two highest coefficients are given in bold.

**Table 2:** Statistical differences of fold change based on delayed breastfeeding initiation.

Populations	Krustal: all bins	Wilcoxon: 0–3, 3−5	Cohen's d: 0–3, 3−5	Wilcoxon: 3–5, 5−23	Cohen's d: 3–5, 5−23	Wilcoxon: 0–3, 5−23	Cohen's d: 0–3, 5−23
Granulocytes	0.001	0.000	2.026	1.000	0.299	0.016	1.538
HLADR+CD14+ Monocytes	0.011	0.001	1.740	0.711	0.067	0.311	0.789
Classical monocytes	0.008	0.003	1.774	0.711	−0.020	0.183	0.823
CD56 Hi NK	0.047	0.163	0.863	0.041	0.000	0.182	0.000
CD11b+CD16+mature neutrophils	0.001	0.001	2.199	1.000	0.332	0.007	1.682
CD11b−CD16+Immature Neutrophils 2	0.001	0.000	2.127	1.000	0.232	0.011	1.503
CD11b+CD16+CD64+	0.000	0.000	1.155	0.711	−0.525	0.007	0.950
CD11b+CD16+CD64−	0.011	0.011	1.371	1.000	0.218	0.059	1.199

Wilcoxon rank-sum test *p*-values, Kruskal–Wallis test *p*-values, and Coehn's d coefficients of significant cell populations (*p* ≤ 0.05, Krustal–Wallis test) for the statistical evaluation of time-to-breastfeeding initiation on fold change (FC = DOL1/DOL0), considering three intervals of hours after birth: 0–3, 3−5, and 5–23.

## Data Availability

The data analyzed in this manuscript are available at ImmPort under accession numbers SDY1538 and SDY2311. All analysis codes have been deposited at https://bitbucket.org/pvp-dmac/epic_hipc_ontogeny/src/master/Flow_Cytometry and are publicly available. flowTypeFilter code and its documentation is available on github under the MIT license at https://github.com/eve-chen97/flowTypeFilter.

## Supplementary Materials

Supplementary Materials Table S1: summary of clinical information. Table S2: list of markers for the Innate and Adaptive Panels. Table S3: data quality control results main cohort. Table S4: data quality control results second cohort. Table S5: statistical evaluation of breastfeeding effects at DOL0. Figure S1: adaptive Panel Gating strategy. Figure S2: innate Panel Gating strategy. Figure S3: control of the batch effect for the Adaptive Panel. Figure S4: control of the batch effect for the Innate Panel. Figure S5: statistical analysis shows no batch effects for the Innate Panel and Adaptive Panel. Figure S6: the 1st criterion of the post-gating quality control step. Figure S7: the 2nd criterion of the post-gating quality control step. Figure S8: flowType algorithm generates all possible combinations of markers phenotypes. Figure S9: flowTypeFilter algorithm can generate non orthogonal gates. Figure S10: PCA analysis considering the flowTypeFilter populations of the Adaptive Panel. Figure S11: variations in cell counts in the first week of life are not impacted by ethnic background. Figure S12: no impact of delayed breastfeeding on cell counts of later fold changes.
