# Supplementary material for: Breastfeeding and Neonatal Age Influence Neutrophil-Driven Ontogeny of Blood Cell Populations in the First Week of Human Life
Source: J Immunol Res. 2024 Jul 23;2024:1117796. doi: 10.1155/2024/1117796 (PMC11288693; doi:10.1155/2024/1117796)
Supplement: Supplementary Materials — Table S1: summary of clinical information. Table S2: list of markers for the Innate and Adaptive Panels. Table S3: data quality control results main cohort. Table S4: data quality control results second cohort. Table S5: statistical evaluation of breastfeeding effects at DOL0. Figure S1: adaptive Panel Gating strategy. Figure S2: innate Panel Gating strategy. Figure S3: control of the batch effect for the Adaptive Panel. Figure S4: control of the batch effect for the Innate Panel. Figure S5: statistical analysis shows no batch effects for the Innate Panel and Adaptive Panel. Figure S6: the 1st criterion of the post-gating quality control step. Figure S7: the 2nd criterion of the post-gating quality control step. Figure S8: flowType algorithm generates all possible combinations of markers phenotypes. Figure S9: flowTypeFilter algorithm can generate non orthogonal gates. Figure S10: PCA analysis considering the flowTypeFilter populations of the Adaptive Panel. Figure S11: variations in cell counts in the first week of life are not impacted by ethnic background. Figure S12: no impact of delayed breastfeeding on cell counts of later fold changes. [file 1117796.f1.pdf]

Supplementary Figures and Tables

	The Gambia (N=756)	PNG (N=40)
**Sex:**		
Male	51%	32%
Female	49%	68%
**Ethnicity:**		
Fula	12%	0%
Jola	15%	0%
Mandika	48%	0%
Serahule	4%	0%
Wollof	11%	0%
Others	10%	0%
Eastern Highland	0%	100%
**Birth Season:**		
Dry Season (November-May)	63%	55%
Rainy season (June-October)	37%	45%
**Breastfeeding (V1):**		
Yes	89%	5%
No	11%	95%
**Maternal age:**		
< 30 years	65%	88%
> 30 years	35%	12%
**Vaccine Group:**		
BCG	24%	0%
HBV	23%	0%
HV+BCG	24%	0%
Delayed	30%	100%
**Birth weight (grams):**		
Mean (SD)	3165.2 (387)	3329.6 (386)
Median [min,max]	3150 [2500,4400]	3300 [2500,4300]

Supplementary Table 1: **Summary of clinical information.** The first and second column of the table indicate, respectively, the percentage of participants with certain clinical attributes (reported along the rows) for The Gambia and Papua New Guinea (PNG) cohort. The letter "N" indicates the number of total participants.

INNATE FLOW CYTOMETRY PANEL	
**TARGET**	**FLUOROCHROME**
CD64	Alexa 700
CD11c	APC
CD123	PE-Cy7
CD3	PE-CF594
gd TCR	PE
CD56	BV650
CD11b	BV786
CD16	FITC
CD45	V450
**ADAPTIVE FLOW CYTOMETRY PANEL**	
**TARGET**	**FLUOROCHROME**
CD10	Alexa 700
CD19	APC
CD27	PE-Cy7
CD38	PE-Cy5
IgD	PE-CF594
CD34	PE
IgM	FITC
CD20	BV786
CD138	V450
**COMMON MARKERS IN INNATE AND ADAPTIVE PANELS**	
**TARGET**	**FLUOROCHROME**
FVD	APC-eFluor780
CD66	Biotin/ BV711 Streptavidine
CD14	V500
HLADR	eFluor605

Supplementary Table 2: **List of markers for the Innate and Adaptive Panels.** The first and second column of the table indicate, respectively, the markers and fluorochromes employed.

QC step	Adaptive Panel	Innate Panel
Total number of samples	1,408 (711 participants)	1,408 (711 participants)
Number of samples after bio-samples QC	1,408 (4 flagged)	1,408 (8 flagged)
Number of samples after post-gating QC	1,382 (710 participants)	1,379 (709 participants)
Excluded samples after post-gating QC	26 (1.8%)	29 (2%)
Excluded samples after metadata QC	24 (1.7%)	23 (1.6%)
Final number of samples analyzed	1,358 (698 participants)	1,356 (697 participants)

Supplementary Table 3: **Data quality control results main cohort**. Data quality control results of each step (first column) for the Adaptive Panel (second column) and Innate Panel (third column).

QC step	Adaptive Panel	Innate Panel
Total number of samples	152 (85 participants)	152 (85 participants)
Number of samples after bio-samples QC	152 (0 flagged)	152 (0 flagged)
Number of samples after post-gating QC	147 (83 participants)	151 (85 participants)
Excluded samples after post-gating QC	5 (3.2%)	1 (0.6%)
Excluded samples after metadata QC	0	0
Final number of samples analyzed	147 (83 participants)	151 (85 participants)

Supplementary Table 4: **Data quality control results second cohort**. Data quality checking results of each step (first column) for the Adaptive Panel (second column) and Innate Panel (third column).

Populations	kruskal:all bins	wilcoxon:0-3,3-5	cohen'd:0-3,3-5	wilcoxon:3-5,5-23	cohen'd :3-5,5-23	wilcoxon:0-3,5-23	cohen'd:0-3,5-23
**Granulocytes**	0.001	0.001	-1.450	0.557	0.140	0.004	-1.010
**HLADR+ CD14+ Monocytes**	0.009	0.002	-1.139	0.370	0.453	0.218	-0.615
**Classical Monocytes**	0.010	0.002	-1.084	0.429	0.412	0.185	-0.626
**Non classical Monocytes**	0.031	0.007	-1.004	0.299	0.611	0.703	-0.353
**CD56-CD16+ NKT cells**	0.017	0.006	-0.975	0.550	-0.005	0.092	-0.668
**CD11b+CD16+ Mature Neutrophils**	0.001	0.000	-1.501	0.615	0.088	0.002	-1.075
**CD11b-CD16+ Immature Neutrophils 2**	0.001	0.001	-1.281	0.466	0.530	0.012	-0.823
**CD11b+CD16+CD64+**	0.004	0.001	-1.390	0.487	0.244	0.079	-0.781

Supplementary Table 5: **statistical evaluation of breastfeeding effects at DOL0**. Wilcoxon Rank-Sum test p-values, Kruskal-Wallis test p-values and Coehn's d coefficients of significant cell populations ($p<0.05$, Krustal Wallis test) for the statistical evaluation of breastfeeding effects on cells/ul across 3 intervals of hours after birth.

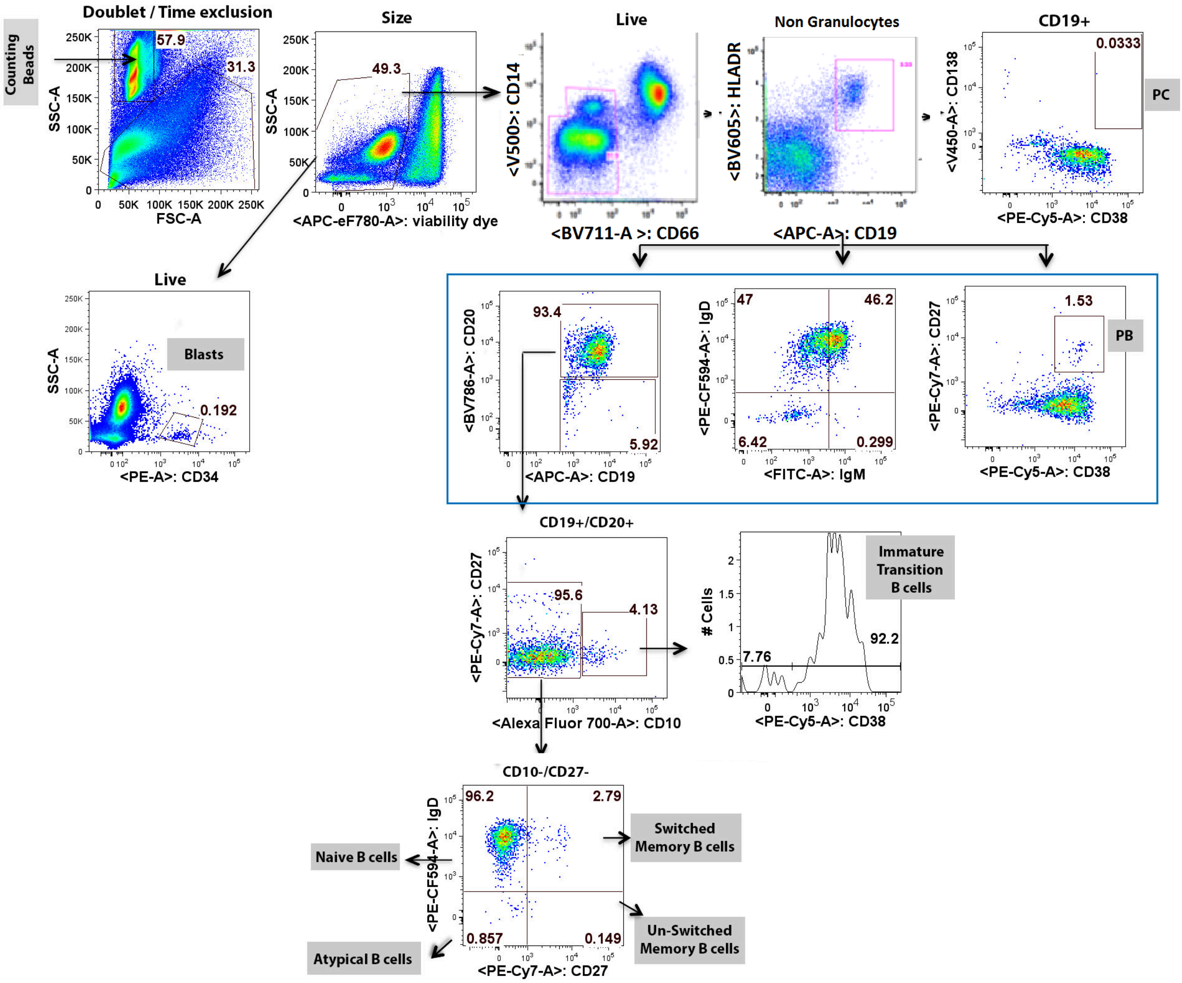

Supplementary Figure 1: **Adaptive Panel Gating strategy**. Each bivariate plot represents a gating step of the hierarchy. The arrows indicate the passage between different steps.

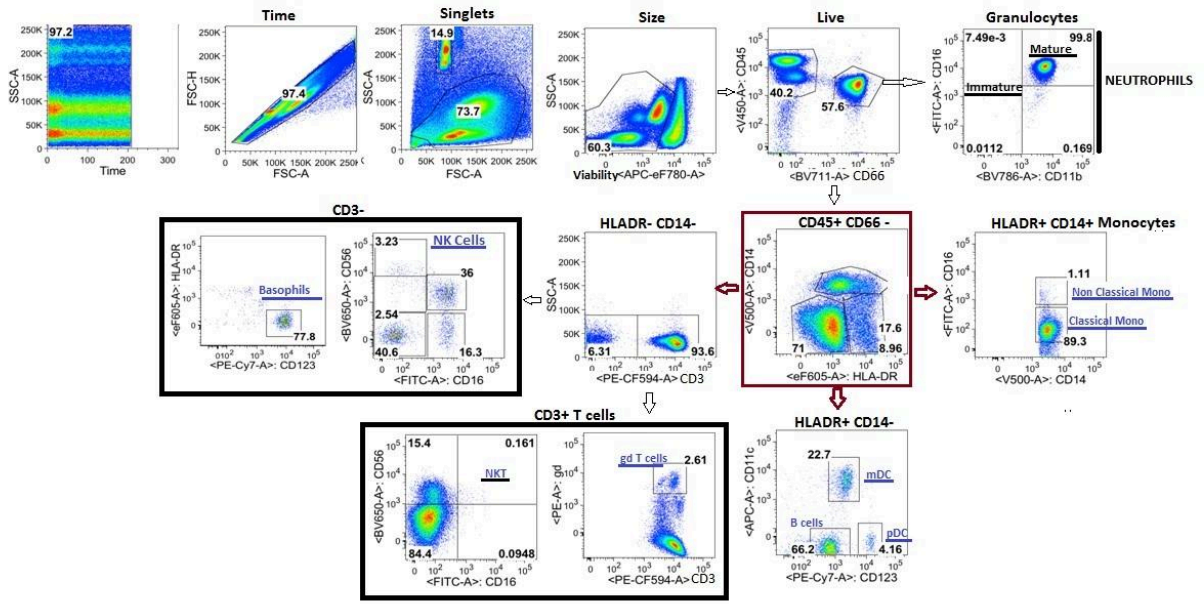

Supplementary Figure 2: **Innate Panel Gating strategy**. Each bivariate plot represents a gating step of the hierarchy. The arrows indicate the passage between different steps.

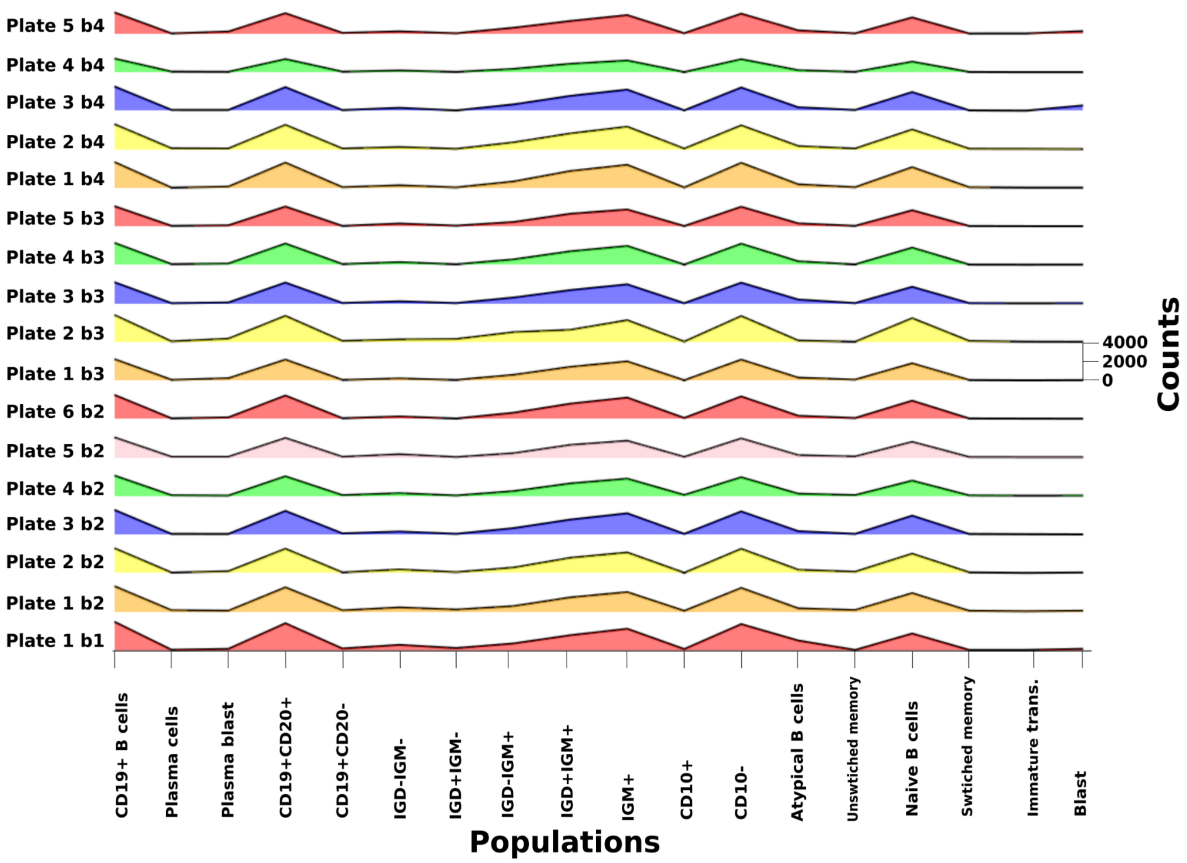

Supplementary Figure 3: **Control of the batch effect for the Adaptive Panel**. Counts of the control samples with plate number (y axis) and population (x axis).

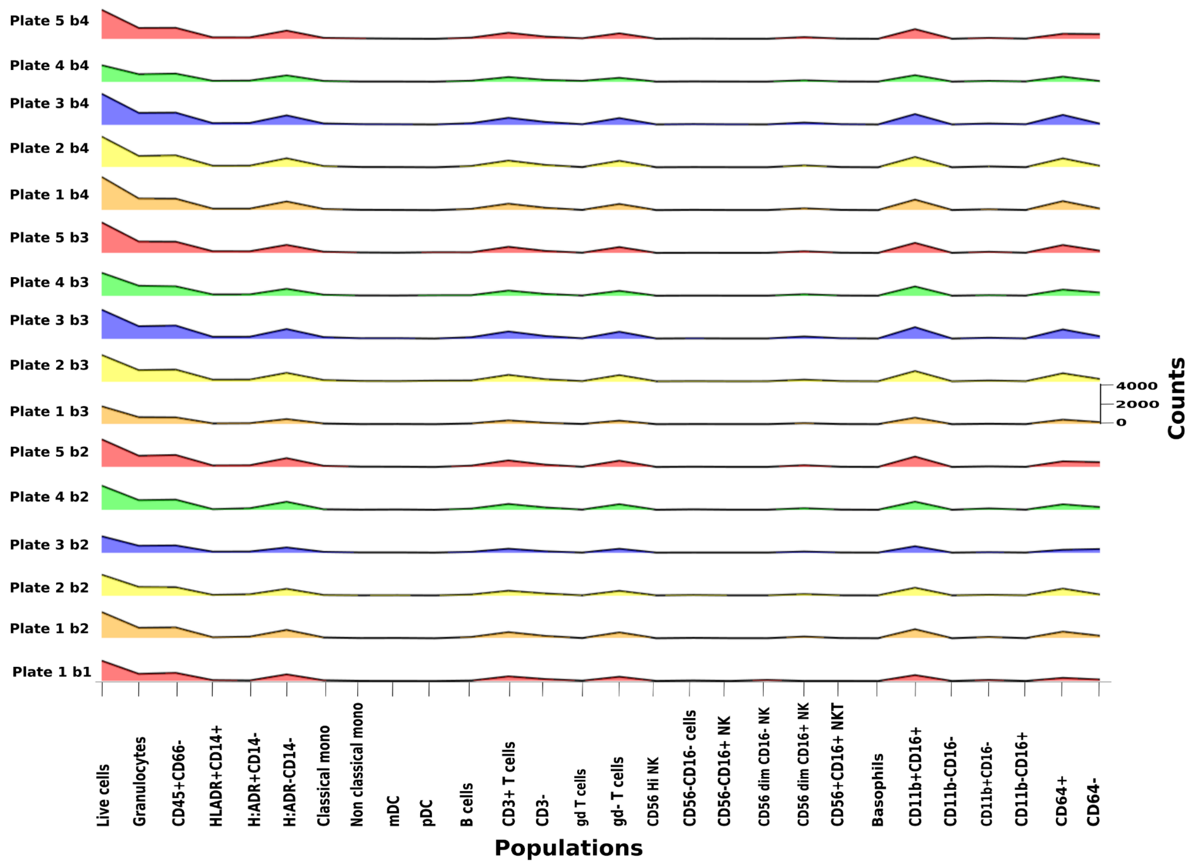

Supplementary Figure 4: **Control of the batch effect for the Innate Panel**. Counts of the control samples with plate number (y axis) and population (x axis).

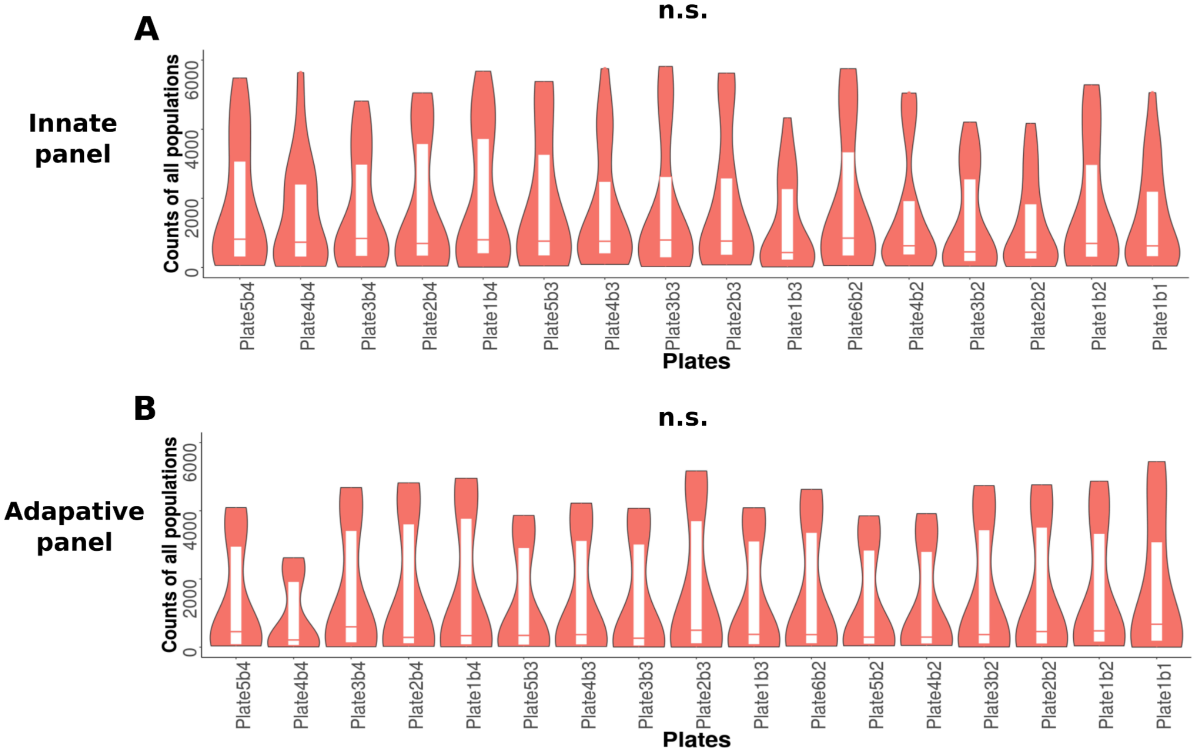

Supplementary Figure 5: **Statistical analysis shows no batch effects for the Innate Panel and Adaptive Panel**. Counts of the control samples of all populations (y axis) across all plates and batches (x axis) in the Innate Panel (A) and Adaptive Panel (B). n.s. $p > 0.05$ by Kruskal-Wallis test.

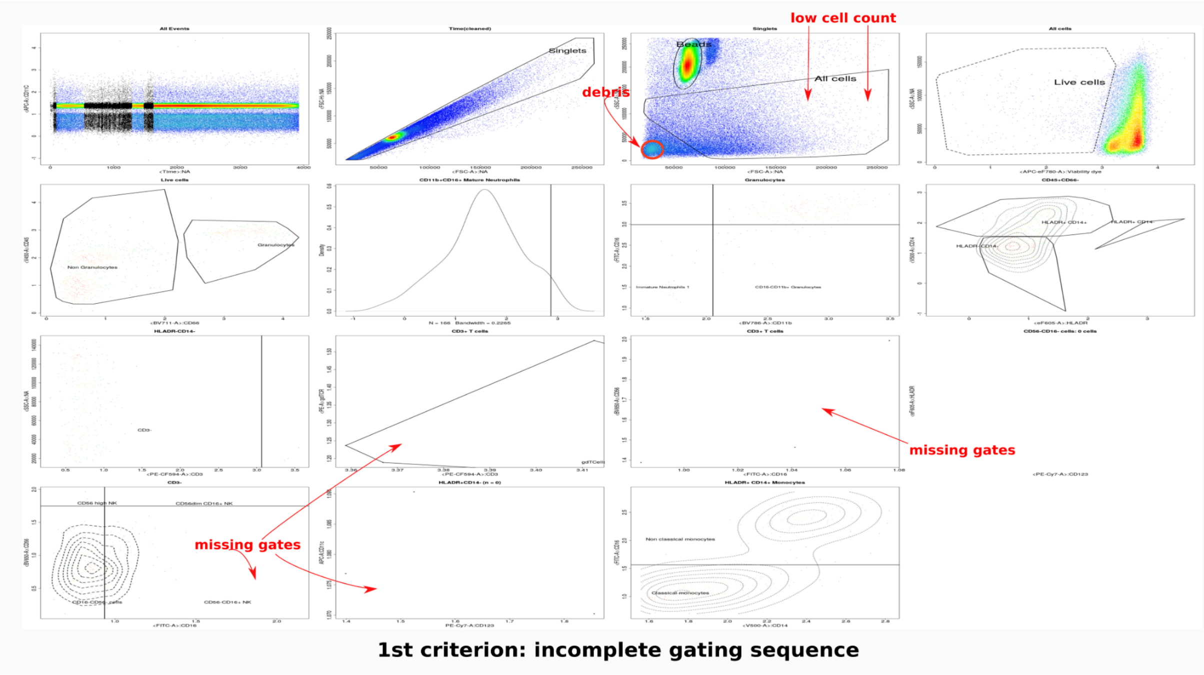

Supplementary Figure 6: **The 1st criterion of the post-gating quality control step.** Gating plot of one sample excluded according to the first criterion (exclusion of samples with incomplete gating sequence due to empty parent populations).

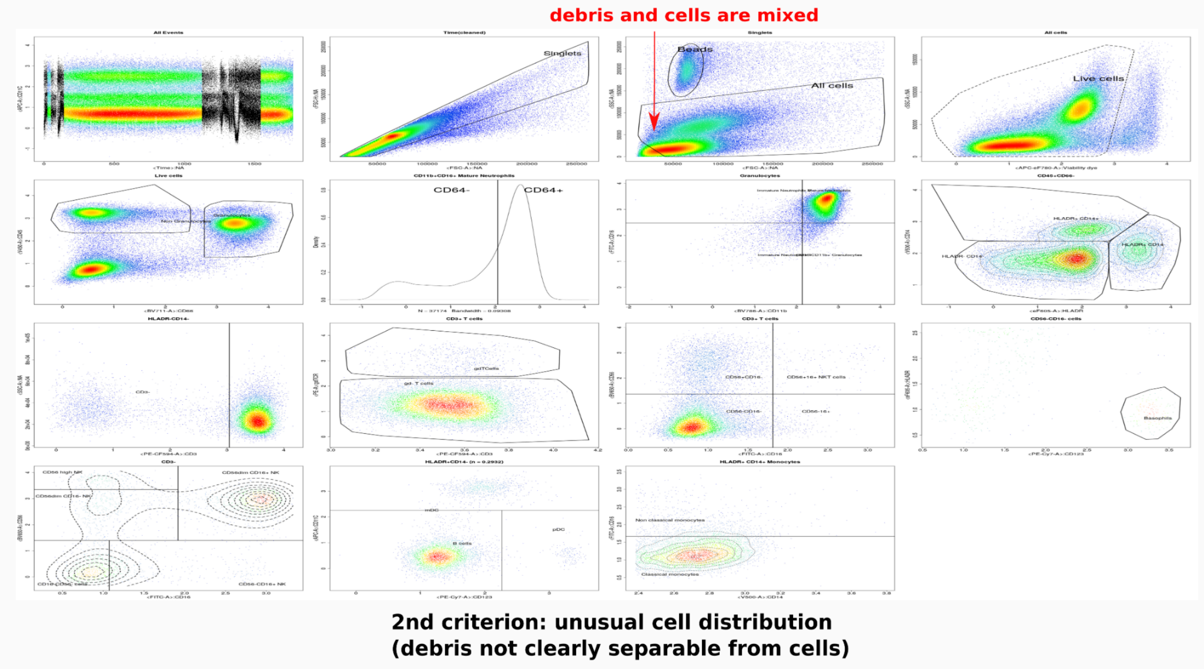

Supplementary Figure 7: **The 2nd criterion of the post-gating quality control step.** Gating plot of one sample excluded according to the second criterion (exclusion of samples with unusual cell distribution).

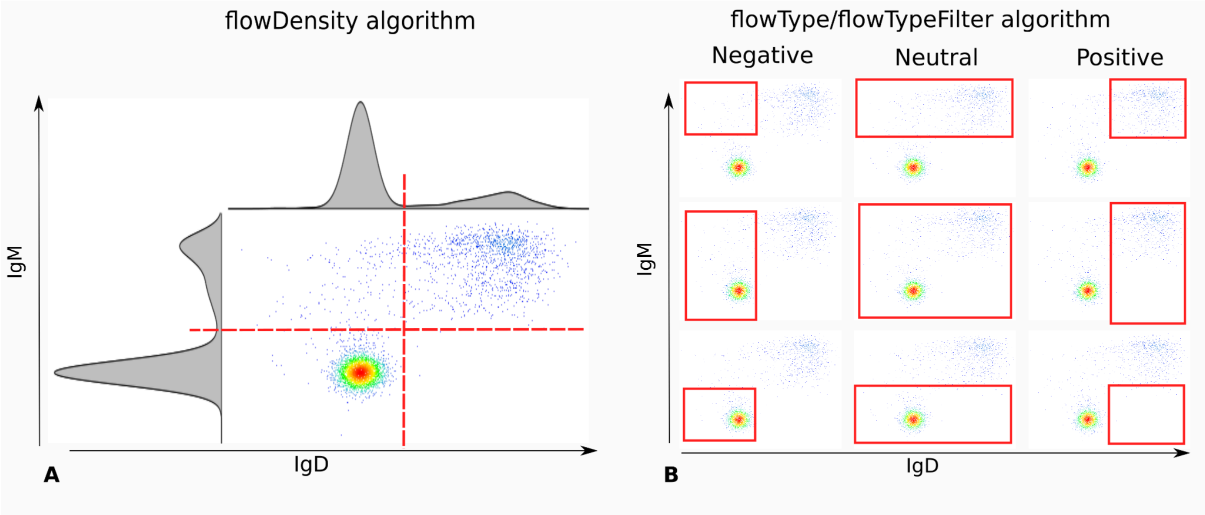

Supplementary Figure 8: **flowType algorithm generates all possible combinations of markers phenotypes.** Comparison between the flowDensity algorithm (A) and the flowType/flowTypeFilter algorithm (B) on the same sample. The red dashed lines represent the thresholds calculated automatically by flowDensity based on the density of the marker expression values. The density of each marker is represented next to each axis and coloured in gray. Red rectangles indicate the gates calculated by flowType or flowTypeFilter based on the flowDensity thresholds.

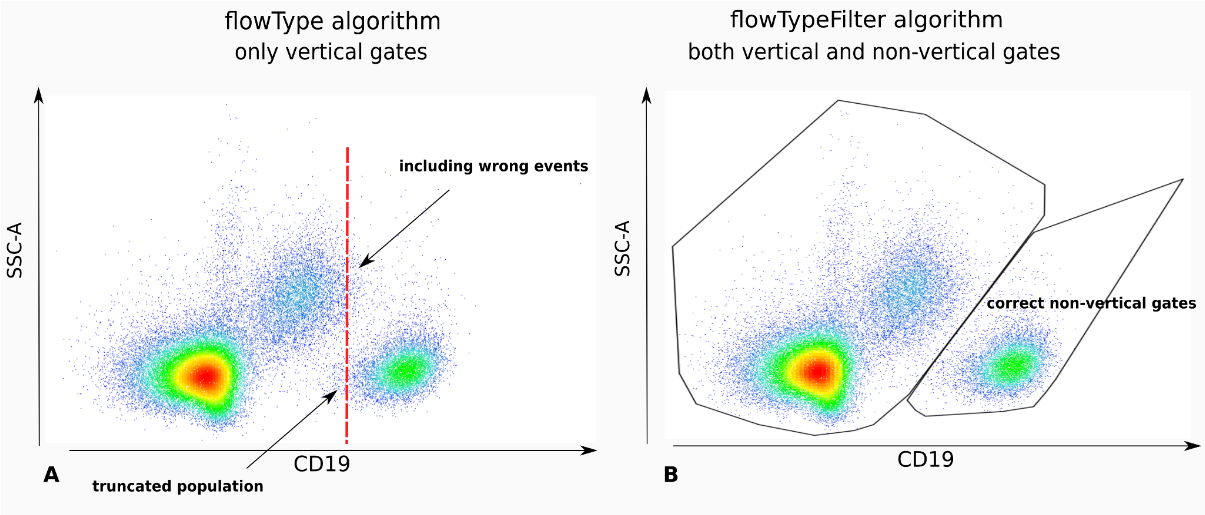

Supplementary Figure 9: **flowTypeFilter algorithm can generate non orthogonal gates**. Comparison between the flowType algorithm (A) and the flowTypeFilter algorithm (B) on the same sample. The red dashed line represents the gate calculated by flowType (based on the flowDensity threshold). The black polygons represent the gates calculated by flowTypeFilter (based on the flowDensity threshold).

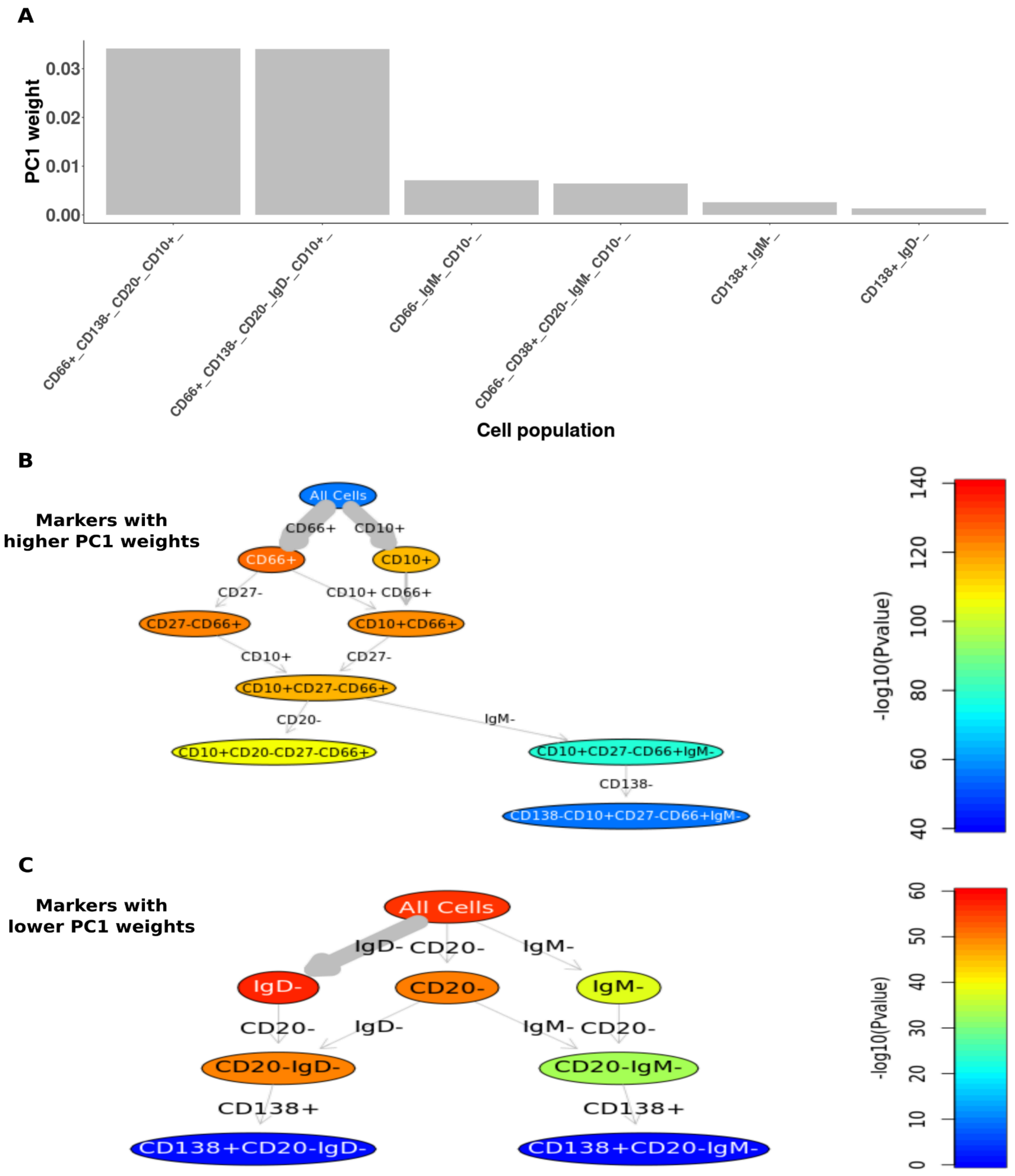

Supplementary Figure 10: **PCA analysis considering the flowTypeFilter populations of the Adaptive Panel**. Weights of PC1 for Adaptive Panel (A); Rchyoptymyx analysis for Adaptive Panel (B,C) with significant immunophenotypes colored in orange-red, not significant immunophenotypes colored in blue.

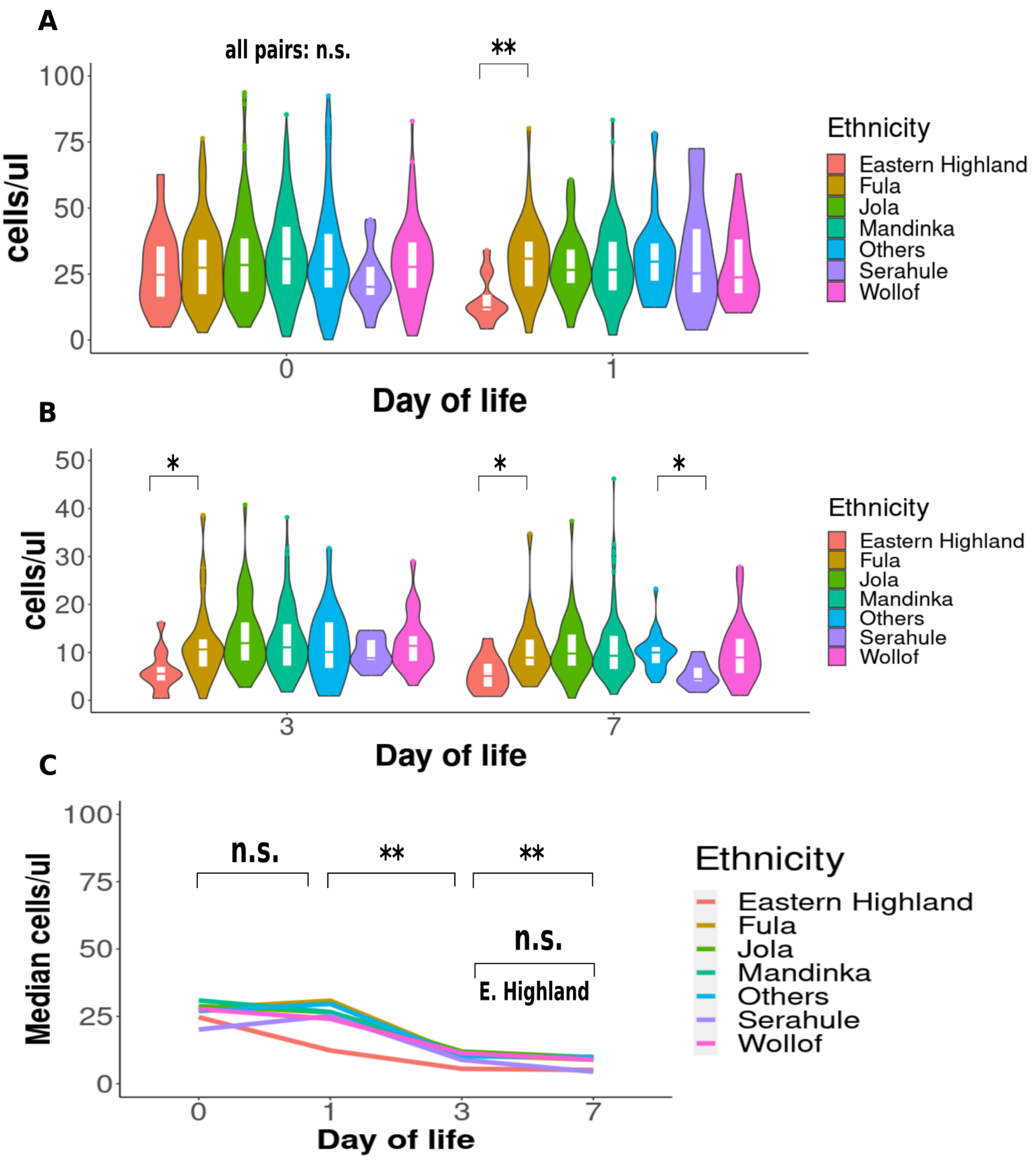

Supplementary Figure 11: **Variations in cell counts in the first week of life are not impacted by ethnic background.** Ethnicities comparison at given time point (A,B); ethnicities comparison across time points (C). ** $p \leq 0.01$, * $p \leq 0.05$ and n.s. $p > 0.05$ by Wilcoxon Rank-Sum test.

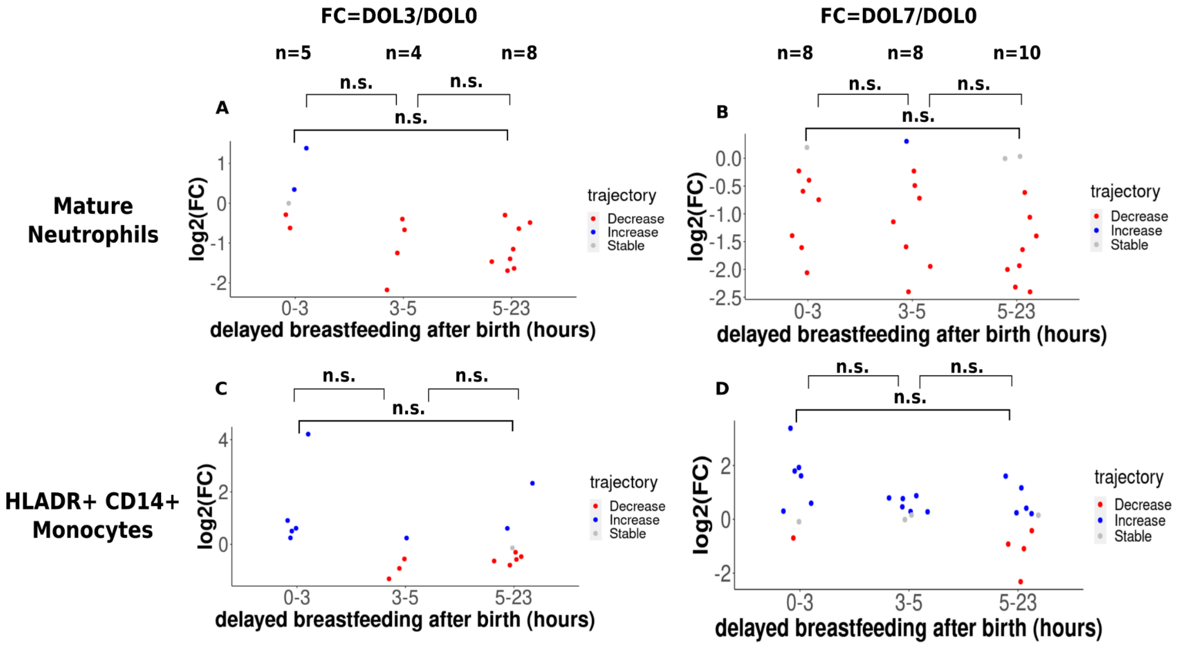

Supplementary Figure 12: **No impact of delayed breastfeeding on cell counts of later fold changes.** Impact of later breastfeeding on peripheral blood mature neutrophil and HLADR+CD14+ monocyte counts across the first 23 hours of life (DOL0) considering later fold changes. (A) Mature neutrophils Fold Change (FC=DOL3/DOL0); (B) Mature neutrophils Fold Change (FC=DOL7/DOL0); (C) Monocytes Fold Change (FC=DOL3/DOL0); (B) Monocytes Fold Change (FC=DOL7/DOL0). n.s. $p > 0.05$ by Wilcoxon Rank-Sum test.
